# The media framing dataset: Analyzing news narratives in Mexico and Colombia

**DOI:** 10.1016/j.dib.2025.111284

**Published:** 2025-01-09

**Authors:** Juan Cuadrado, Elizabeth Martinez, Juan Carlos Martinez-Santos, Edwin Puertas

**Affiliations:** Universidad Tecnológica de Bolívar, Colombia

**Keywords:** Computational linguistics, Media analysis, Cross-cultural studies, News content, Sentiment analysis, NLP resources, Content annotation

## Abstract

This paper introduces “The Media Framing Dataset,” a dataset developed through an in-depth examination of news articles from 140 local newspapers in Mexico and Colombia, covering events from May 2022 to August 2023. Our dataset captures a broad spectrum of topics, including politics, immigration, public opinion, and crime. The data collection involved a meticulous keyword-based search strategy designed to identify articles that illustrate various news-framing dimensions, such as Economics, Policy, Morality, and more.

To construct this dataset, we employed a combination of manual and automated annotation techniques. Articles were categorized based on specific framing dimensions using a structured framework, developed in collaboration with experts in computational linguistics. The annotation process, conducted by trained annotators from Mexicoʼs Delfin program, guarantees both precision and depth.

“The Media Framing Dataset” serves as a valuable resource for NLP research with high potential for reuse. It is particularly suitable for analyzing cultural and linguistic nuances in media framing, assessing the impact of framing on public perception, and supporting the development of models that automatically detect framing techniques. Additionally, it provides a foundation for linguistic analysis and machine learning projects, enabling researchers and practitioners to explore media framing dynamics and develop innovative tools for media analysis.

Specifications Table

**Table:** 

Subject	Media Technology.
Specific subject area	Multidimensional media framing analysis via computational linguistics.
Data format	Raw and Analyzed.
Type of data	A table (CSV format) and folder (TXT files).
Data collection	Data were collected via web scraping using the search engines of El Tiempo in Colombia and El Universal in Mexico, targeting a total of 140 labeled articles from May 2022 to August 2023. Articles were chosen for their broad national audience and general content scope. The annotation process involved manual labeling on the Labelbox platform, focusing on accurately capturing diverse media framing dimensions.
Data source location	*“El Tiempo”* - Colombia; *“El Universal”* - Mexico.
Data accessibility	**Repository name:** Zenodo **Data identification number:** 10909879 **Direct URL to data:**https://zenodo.org/record/10909879 **Instructions for accessing these data:** Access the dataset by visiting the provided URL. The data is publicly available and can be downloaded directly from Zenodo. Anonymous access is enabled for editors and reviewers.

## 1 Value of the Data

• *Insight into Media Framing Practices:* This dataset facilitates exploration of how news media in Colombia and Mexico might frame societal issues, potentially reflecting certain cultural and political contexts. It serves as a resource for those interested in examining regional media practices and their possible impacts on society.
• *Broad Applicability for Researchers:* Beneficial for researchers in media studies, sociology, political science, and computational linguistics, this dataset supports investigations into news framing effects and discourse within Latin America.
• *Tool for Developing NLP Applications:* Ideal for developing NLP tools to detect media framing, analyze sentiment, and categorize content, enhancing computational linguistics research.
• *Potential for Comparative Insights:* This dataset, which includes articles from both Colombia and Mexico, may provide a basis for exploring how media framing practices might differ between these two countries. It offers researchers a starting point to examine potential variations in media coverage related to cultural and political environments.
• *Educational Resource:* Useful as a teaching tool in academic settings to demonstrate media framing processes, its impact, and the application of NLP techniques in analyzing real-world media.
• *Enhancement of Automated Media Monitoring Tools:* The dataset aids in improving the functionality and accuracy of automated media analysis systems, suitable for developers and researchers enhancing media monitoring technologies.

## 2 Background

**1. Regional Contexts: Mexico and Colombia**

Focusing on Mexico and Colombia, regions often underrepresented in global media studies, presents a unique opportunity to explore how local contexts influence media narratives. These regions are characterized by their vibrant political landscapes and cultural diversity, which are frequently reflected in media framing practices. This study leverages a dataset specifically curated from major newspapers like “El Universal” and “El Tiempo,” providing a nuanced understanding of how regional issues are framed and discussed within these communities. The dataset developed through this study, detailed in “Framing Across Borders: News Media Corpus Framing in Mexico and Colombia,” serves not only as a substantial academic resource but also as a foundation for future research [1].

**2. Media Framing and Its Significance**

Media framing is a critical concept in communication studies, encapsulating how media coverage shapes public perception by emphasizing specific attributes of news stories. Framing influences how audiences interpret events and issues, significantly impacting public opinion and societal discourse. Robert M. Entman's seminal work highlights that framing effectively selects and saliently highlights certain aspects of reality, thereby shaping the narrative in a way that can align with specific agendas or perspectives [2]. This power of framing makes it a pivotal area of study for scholars aiming to understand the mechanics of media influence across different cultural and political landscapes.

**3. Technological Advancements in Media Analysis**

Recent years have seen significant advancements in natural language processing and machine learning [3], which have been applied to automate and refine the analysis of media framing. These technologies enable researchers to dissect complex narrative structures and understand the subtleties of tone and context at a granular level. For instance, the works by Piskorski et al. [4] and Liu et al. [5] demonstrate how multi-lingual setups and advanced computational techniques can be used to detect framing and persuasion techniques, offering insights into the adaptability and application of these computational methods across languages and regions.

**4. Evolution of Media Framing Analysis**

Historically, media framing research has relied heavily on qualitative analyses, which, while insightful, were often limited by the scale and speed of manual annotation. The advent of computational tools has revolutionized this field, allowing for the analysis of large datasets with greater accuracy and efficiency. Studies like those conducted by Card et al. [6] and Kwak et al. [7] have employed these tools to analyze thousands of articles, uncovering patterns and trends in framing that were previously unattainable. Further enriching the field, the guidelines by Piskorski et al. [8] have set new standards for categorizing and understanding media frames, enhancing the precision and applicability of research methodologies in media studies.

## 3 Data Description

This dataset, titled *“Framing Across Borders: News Media Corpus Framing in Mexico and Colombia,”* comprises 140 labeled articles from two prominent news outlets, El Tiempo in Colombia and El Universal in Mexico. The dataset is structured as follows:

1. **File Structure:**
  • [labels.csv]: Summarizes labels and other metadata associated with each article.
  • [articles.zip]: Contains a folder with the text files for each article's content.
2. **Data Format:**
  • Each article is stored as a UTF8-encoded text file:
  • The title appears on the first row of each file.
  • The content of the article starts from the second row onwards.
  • Files are named using the format: Noticia_{ID}.txt, where {ID} is a unique numerical identifier, e.g., Noticia_1.txt.
3. **CSV File Details**

The CSV file contains detailed metadata and labeling information for each article:

• **ID:** Unique article ID.
• **Frames:** Framing dimensions applied to the article.
• **Tone:** Overall tone of the article.
• **Principal_frames:** Primary framing dimensions.

## 4 Experimental Design, Materials and Methods

This study designed a methodology aimed at exploring media framing within the specific contexts of Mexico and Colombia. The process outlined below adheres to a chronological and logical sequence, ensuring both replicability and depth in the analysis:

1. **Preliminary Research and Conceptual Definition:**

The project commenced with a comprehensive review of existing literature on media framing, focusing on its principal dimensions and the nuances of annotation processes. This phase revealed that news articles typically exhibit framing and tonality at both paragraph and overall article levels, leading to the refinement of the framing dimensions employed in our annotations, as detailed in Table 1.

**Table 1:** Framing dimensions definition and examples.

Framing dimension	Definition	Example context
Economic	Costs, benefits, or other financial implications.	Articles discussing the economic impact of immigration policies.
Capacity and Resources	Availability of physical, human, or financial resources, and capacity of current systems.	Coverage on healthcare system capacities during a pandemic.
Morality	Religious or ethical implications.	Debates surrounding abortion laws.
Fairness and Equality	Balance or distribution of rights, responsibilities, and resources.	Discussions on income inequality and social justice.
Legality, Constitutionality, and Jurisprudence	Rights, freedoms, and authority of individuals, corporations, and government.	Legal analyses of new legislation affecting civil liberties.
Policy Prescription and Evaluation	Discussion of specific policies aimed at addressing problems.	Opinions on climate change mitigation strategies.
Crime and Punishment	Effectiveness and implications of laws and their enforcement.	Reporting on criminal justice reform efforts.
Security and Defense	Threats to the welfare of the individual, community, or nation.	National security concerns related to border control.
Health and Safety	Health care, sanitation, public safety.	Measures to combat the spread of infectious diseases.
Quality of Life	Threats and opportunities for the individual's wealth, happiness, and well-being.	The impact of urban development on residents' well-being.
Cultural Identity	Traditions, customs, or values of a social group with a policy issue.	Preservation of indigenous languages and cultures.
Public Opinion	Attitudes and opinions of the general public, including polling and demographics.	Surveys on public support for renewable energy sources.
Political	Considerations related to politics and politicians, including lobbying, elections, and attempts to sway voters.	Election campaign strategies and voter mobilization efforts.
External Regulation and Reputation	International reputation or foreign policy.	Diplomatic relations and international agreements.
Other	Any coherent group of frames not covered by the above categories.	Unique or emerging frames specific to contemporary issues.

**2. Dataset Generation**

To create a comprehensive dataset for the study, we employed advanced web scraping techniques using Python, specifically leveraging the BeautifulSoup library. We collected a total of 140 articles from two prominent local newspapers, *"El Universal"* in Mexico and *"El Tiempo"* in Colombia. These articles were selected using a carefully constructed keyword search strategy. The keywords were chosen to capture a broad range of themes and perspectives relevant to various framing dimensions, such as *"economic impact"* and *"public health"* (see Table 1 for detailed examples).

The scraping process extracted article metadata including headlines, body text, publication dates, authors, and URLs. To ensure a high-quality dataset, we implemented filtering mechanisms to exclude irrelevant content, such as advertisements, using regex and HTML parsing techniques. Duplicates were removed by comparing URLs and titles. The cleaned data was formatted into .txt files and organized in a structured format, preserving essential metadata. These files were then prepared for annotation by uploading them to the Labelbox platform.

**3. Dataset Scope and Context**

The dataset includes 140 articles from two prominent newspapers, El Universal in Mexico and El Tiempo in Colombia. This sample size was carefully chosen to balance depth and quality, ensuring that each article could undergo a rigorous dual-layer annotation process with expert validation. This approach prioritizes reliability and provides deeply annotated examples suitable for meaningful analysis in the context of media framing studies.

Focusing on Mexico and Colombia was a deliberate decision, as the annotators, native speakers from these regions, contributed valuable cultural and linguistic insights. This enhances the dataset's precision and makes it particularly relevant for exploring framing practices in these countries.

While this dataset provides a strong foundation for analyzing media narratives in Latin America, its design allows for future expansions to include additional countries, offering a broader regional perspective and enriching comparative research in this field.

**4. Development of the Annotation Protocol**

A rigorous annotation protocol was developed to ensure consistency and reliability across the dataset annotations. This protocol was constructed based on best practices from existing studies on media framing, including guidelines from [6,7]. The protocol covered the following key elements:

1. Basic Data Identification: Annotators were instructed to accurately log metadata (date, title, author, text body) for each article.
2. Paragraph-Level Tone and Frame Analysis: Each paragraph was assessed individually for both framing dimension and tone, following the categories detailed in Table 1*.*
3. Global Tone and Frame Determination: Annotators determined the predominant tone and frame for the entire article based on the most frequently occurring categories.
4. Annotation Review and Validation: An iterative review process was implemented, involving senior reviewers who assessed each annotation against the protocol criteria. Feedback was provided continuously to ensure annotator alignment with the protocol.

**5. Annotator Training**

The annotators were undergraduate engineering exchange students from the DELFIN program at UTB. Their training program, lasting two weeks, was comprehensive and designed to ensure consistent application of the annotation framework. Training included:

• Lectures and Workshops: Conducted in collaboration with faculty from the Schools of Communications, Systems Engineering, and AI-specialized Computer Science.
• Hands-on Practice: Annotators participated in guided annotation sessions on Labelbox, using practice datasets and receiving real-time feedback.
• Evaluation and Feedback: Regular evaluations through quizzes and practical exercises to gauge understanding, followed by feedback sessions to address gaps.
• Final Assessment: A certification test ensuring each annotator met the required proficiency level before starting the actual annotation tasks.

**6. Annotation Process on Labelbox**

The annotation process was conducted on the Labelbox platform, which was customized with a schema specifically designed for this project. The schema, based on an ontology of frames and tones, included all framing dimensions detailed in Table 1. The process was structured as follows:

• Assignment of Articles: Each of the 140 articles was randomly assigned to two annotators to ensure a diverse range of perspectives.
• Dual-Layer Review: Both annotators reviewed each article independently. Discrepancies greater than 30 % between annotations were flagged for further review.
• Final Validation: Senior annotators reviewed flagged articles, and a final decision was made by an expert reviewer to ensure adherence to the annotation protocol.

**7. Data Extraction and Processing**

Following the completion and validation of the annotations, the data was exported from Labelbox in JSON format and converted to CSV for analysis. Each article was preserved in UTF-8 encoded .txt files, with unique identifiers *(*e.g.*, “Noticia_{ID}.txt”)* for traceability. The annotated data was organized into a structured format suitable for machine learning applications and further analysis, such as NLP-based studies on media framing.

**8. Quality Assurance and Data Integrity**

To maintain high standards of data quality and integrity, a multi-layered quality assurance process was implemented:

• Regular Checks: Automated scripts checked for missing data, inconsistencies, and format errors.
• Inter-Annotator Agreement: The consistency between annotators was evaluated using metrics like Krippendorff's alpha, with a minimum threshold set for acceptable agreement levels[6,7].
• Consensus Discussions: Annotators participated in regular consensus meetings to resolve discrepancies and align their interpretations.
• Continuous Feedback: Annotators received continuous feedback from senior reviewers to improve their performance and maintain adherence to the protocol.

**9. Software and Tools**

The study utilized the following software and tools:

• Python Version: 3.8
• Libraries:
  ○ BeautifulSoup: For web scraping and HTML parsing.
  ○ Pandas: For data manipulation and conversion between formats (e.g., JSON to CSV).
  ○ NLTK (Natural Language Toolkit): For preprocessing textual data.
  ○ Matplotlib: For data visualization to assess the distribution and characteristics of frames and tones.
• Labelbox: Used for annotation management, custom schema design, and inter-annotator agreement analysis.
• GitHub Repository: All scripts for scraping, processing, and analysis were stored in a public GitHub repository to ensure transparency and reproducibility.

## Limitations

The scale of data collection in this study was influenced by several logistical factors. Primarily, the project utilized a team of summer course students for annotations, which was effective but limited by the academic calendar. Once the course concluded, the annotation process also ended, resulting in a dataset that encompasses 140 articles. This time-bound nature of the project was the primary reason for the fixed size of the dataset, rather than an ongoing effort that could have allowed for continuous expansion.

The dataset was also limited to articles from two major newspapers, “El Universal” in Mexico and “El Tiempo” in Colombia. This selection was due to the focus on creating a manageable and cohesive dataset that could be thoroughly annotated within the available timeframe. While this approach ensured a high level of detail and consistency in the annotations, it also constrained the variety of news sources included in the study.

Furthermore, the availability of annotators and the structured timeline of the project primarily restricted the expansion of the dataset. These logistical challenges delineated the scope of the project, underscoring the need for extended operational periods and potentially broader engagement with additional news sources in future research to enhance the dataset's coverage and diversity.

## Ethics Statement

The authors confirm that they have read and adhered to the ethical requirements for publication in Data in Brief. This research did not involve human subjects, animal experiments, or data collected from social media platforms. The data used in this study were obtained through paid subscriptions to established news outlets, and all content was accessed and used in compliance with the respective publication's policies and copyright laws. No ethical approval was required as the data collection involved publicly available information.

## CRediT Author Statement

Conceptualization, Juan Cuadrado; Edwin Puertas; Juan Carlos Martinez-Santos.

Methodology, Juan Cuadrado; Edwin Puertas; Juan Carlos Martinez-Santos.

Software, Juan Cuadrado.

Validation, Edwin Puertas; Juan Carlos Martinez-Santos.

Formal analysis, Juan Cuadrado; Elizabeth Martinez; Edwin Puertas; Juan Carlos Martinez-Santos.

Investigation, Juan Cuadrado; Elizabeth Martinez.

Resources, Juan Cuadrado; Edwin Puertas; Juan Carlos Martinez-Santos.

Data Curation, Victor Pacheco; Daniel López; Sebastián Medina; Denilson Dominguez; Estefanía Marmolejo; Juan Duran; Edwin Montoya; Leopoldo López; Valeria Gutiérrez; Gerardo Muñoz; Maria Fragoso; Miguel De Robles; Diego De La Concha; Rafael Lara; Julian Alcibar-Zubillaga.

Writing - Original Draft, Juan Cuadrado; Elizabeth Martinez.

Writing - Review & Editing, Juan Cuadrado; Elizabeth Martinez; Edwin Puertas; Juan Carlos Martinez-Santos.

Visualization, Elizabeth Martinez.

Supervision, Edwin Puertas; Juan Carlos Martinez-Santos.

Project administration, Edwin Puertas; Juan Carlos Martinez-Santos.

Funding acquisition, none.

## Data Availability

ZenodoFraming Across Borders: News Media Corpus Framing in Mexico and Colombia (Original data).
